# Is the screening able to lower morbidity in the territory?

**DOI:** 10.1186/1748-7161-8-S2-O5

**Published:** 2013-09-18

**Authors:** Aulisa Angelo Gabriele, Vincenzo Guzzanti, Francesco Falciglia, Marco Giordano, Marco Peruzzi, Aulisa Lorenzo

**Affiliations:** 1 Orthopaedic Department, Children's Hospital Bambino Gesù, Institute of Scientific Research, Rome, Italy

## Background

Although several procedures for treating scoliosis have evolved, the most effective treatment is still based on early detection. For early diagnosis of idiopathic scoliosis, many authors proposed methods of school screening; however, a standardized screening program does not yet exist.

## Purpose

The aim of this study was to evaluate a school screening method and the prevalence and distribution of scoliosis in Italian schoolchildren, aged 9-14 years, and to determine if the screening method can reduce morbidity in the territory.

## Methods

The screening program was based on three steps:

A clinical examination was performed by the school physician and two specialists.

Doubtful cases (presence of hump between the two sides of the torso as measured in the thoracic or thoracolumbar region with use of a humpmeter) were evaluated by an orthopedic specialist that prescribed clinical control every six months or X-ray examination.

Classification of scoliosis and procedures for treatment.

All patients were scheduled for a follow-up program and were evaluated in the subsequent three years. Statistical analyses were performed with GraphPad Prism 6.

## Results

A total of 8,995 children were screened for scoliosis. Of the total screened, 487 showed clinical signs of scoliosis and, of these, 181 were referred for antero-posterior radiographs because they had a positive result on the forward-bending test (hump>5mm). No significant statistical difference was observed by the three clinical examiners. Of the 181 patients who were referred, 69 were X rayed, and a clinical diagnosis confirmation was made in 94.2% of the cases. The prevalence of scoliosis (defined as a curve of 10° or more) was 0.76% (65 of 8,995 children), and most of the curves (44; prevalence 67.69%) were small (<20°). The ratio of boys to girls was 1:3.3 overall, but varied according to the magnitude of the curve (1:3 for curves of <20°, 1:3.25 for curves of 20-29° and 1:4 for curves of 30° or larger). Double curves were the most common type identified, followed by thoracolumbar curves; specifically, of the 65 children who had a curve, 21 (32.30%) had a double curve, 18 (27.6%) had a thoracolumbar curve, 17 (26.1%) had a lumbar curve and 9 (13.84%) had a thoracic curve. In the following three years, only four patients were found to have curves >20° and none >30°.

## Conclusions and discussion

Our results prove that the school screening program was accurate and repeatable. Moreover, screening children for scoliosis using a simple test appears to be an effective means of early detection. Above all the screening process effectively decreased the morbidity in the territory at a negligible cost.
